# A Systematic Review of the Use of Routine Versus Selective Episiotomy for Vaginal Birth

**DOI:** 10.7759/cureus.65865

**Published:** 2024-07-31

**Authors:** Nour A Alrida, Amal Ababneh, Khawla Al-Sharif, Diana Arabiat, Jafar Alshraidah, Basheer Al-Zu'bi

**Affiliations:** 1 Nursing, Yarmouk University, Irbid, JOR; 2 Nursing, Jerash University, Jerash, JOR; 3 Critical Care, Jordanian Ministry of Health, Amman, JOR; 4 Maternal and Child Health Nursing, Faculty of Nursing, University of Jordan, Amman, JOR; 5 Clinical Research and Innovation, School of Nursing and Midwifery, Edith Cowan University, Joondalup, AUS; 6 Nursing, University of Jordan, Amman, JOR; 7 Critical Care, Irbid University College, Al-Balqa Applied University, Al-Salt, JOR

**Keywords:** episiotomy, routine episiotomy, selective episiotomy, systematic review, vaginal birth

## Abstract

Episiotomy is a common obstetric surgical procedure involving an incision to enlarge the vaginal opening, facilitating the delivery of the fetus during the second stage of labor. Hence, the current review was conducted to assess the impact of using selective versus routine episiotomy during vaginal birth on birth outcomes. This systematic review used the Joanna Briggs Institute (JBI) methodology for systematic reviews and research syntheses. PubMed, the Cochrane Library, Cumulative Index to Nursing and Allied Health Literature (CINAHL), and SCOPUS were searched for relevant studies. Two reviewers independently screened and extracted data from relevant studies. Four studies met the eligibility criteria and were included in this review. The findings suggest selective episiotomy is associated with better maternal and fetal outcomes than routine episiotomy in certain contexts. However, results varied, indicating the importance of tailoring episiotomy practices to specific patient populations and healthcare settings. To conclude, the review supports the use of selective episiotomy over routine episiotomy during vaginal birth, as it is associated with fewer adverse maternal outcomes.

## Introduction and background

Episiotomy is a common surgical intervention during the second stage of labor [1]. Its prevalence ranges from 11.6% in the United States to 17% in Canada [2]. The Middle East reports a higher prevalence of 67% [3]. This is higher than the recommended percentage by the World Health Organization [4], exceeding the WHO's recommended rate of no more than 10% [4]. High episiotomy rates are often linked to primiparity, oxytocin use, instrumental delivery, perineal tears, and short birth intervals [1,3]. Other factors include fetal distress, meconium-stained amniotic fluid, shoulder dystocia, and macrosomia (≥ 4,000 g) [5].

Obstetricians and gynecologists often use episiotomy to prevent severe perineal tears [6]. While minor tears may heal without intervention, severe tears require surgical repair. The rationale for episiotomy is the prevention of complications such as acute pain, dyspareunia, prolonged hospital stays, urinary and fecal incontinence, fistulas, and pelvic organ prolapse [7]. Ragusa et al. state that episiotomy facilitates labor by enlarging the vaginal outlet, potentially reducing pelvic floor stretching and third and fourth-degree tears [8]. It may also shorten labor by 15-20 minutes, which is crucial in cases of fetal distress, maternal exhaustion, or shoulder dystocia [4].

The literature suggests that healthcare professionals' autonomy influences episiotomy rates [8-10]. Significant disparities in episiotomy rates are linked to the backgrounds of obstetricians and midwives. For example, regional differences in Ireland and Canada and higher rates in the East Midlands compared to Northeastern and Merseyside in the UK illustrate this variation [11,12]. These variations highlight the influence of healthcare providers' preferences, shaped by regional and cultural backgrounds [9].

Conversely, episiotomy can lead to extended tears, acute pain, especially if the episiotomy is mediolateral, and high rates of dissatisfaction among mothers [13]. This is in addition to the risk of postpartum bleeding that can be associated with adverse effects on mothers’ well-being [14]. In the long term, episiotomy may impact muscle integrity and result in permanent weakness at the site of the incision [8]. However, the earlier claim that episiotomy may prevent muscle laxity in the pelvic floor after the birth of a baby was not supported by any practical or theoretical evidence [14].

There was one Cochrane review by Jiang et al. that focused on the effects on mother and baby of a policy of selective episiotomy compared with a routine episiotomy for vaginal birth. The review concluded that selective episiotomy policies resulted in less perineal and vaginal trauma in women where no instrumental delivery is needed. However, it was less clear if routine episiotomy is helpful for women where instrumental delivery is needed [15].

Considering the increased quality of more recent midwifery research, an updated review is needed to cover the literature published from 2016 to 2023 and better inform policymaking and midwifery practices, especially given the advancement in midwifery research over recent years. This period has seen increased rigorous studies, leading to more reliable findings. By integrating these new insights, an updated review will better inform policymakers and midwifery practices. In this review, we review the cumulative evidence on the impact of using selective episiotomy (only in exceptional cases with justification to implement episiotomy) versus routine episiotomy (application of episiotomy to all normal vaginal births without exception) on birth outcomes (perineal tears, blood loss, pain scores, APGAR score, and admission to NICU). By synthesizing the most recent and high-quality research, this review will offer updated, evidence-based recommendations that can significantly enhance clinical guidelines and improve maternal and neonatal care practices.

## Review

Review question

During vaginal birth, what is the impact of using selective versus routine episiotomy on birth outcomes within the immediate postpartum period?

Methodology

After PROSPERO registration (CRD42023446298), scanning for the studies was performed using four databases. Studies reporting maternal and fetal outcomes of pregnant mothers with selective or routine episiotomy were included. Data were screened and extracted independently by two reviewers. The current review followed the Preferred Reporting Items for Systematic Review and Meta-Analyses (PRISMA) guidelines for systematic review of experimental studies [16]. To ensure the study's rigor and quality, we used the Joanna Briggs Institute (JBI) methodology for conducting systematic reviews, appraising the quality, and synthesizing the evidence [17].

Information sources

An electronic search for PubMed, SCOPUS, Cochrane Library, and Cochrane Library, Cumulative Index to Nursing and Allied Health Literature (CINAHL) databases for primary and peer-reviewed research articles. The search was undertaken between July and August 2023 using selective keywords. Then, search manually for the reference list of the included studies to find additional relevant research articles.

Search strategy

A search strategy with the keywords “pregnancy OR pregnant OR maternal outcomes OR birth,” “selective episiotomy OR routine episiotomy” “selective episiotomy” AND “routine episiotomy” OR “optional episiotomy” OR “restrictive episiotomy” was initiated. Then, reference sections were searched manually to find relevant articles (but none were eligible for the review). The reviewers (N.A. and A.A.) screened the abstracts and titles of the articles independently for all retrieved studies. Studies with relevant abstracts and titles were screened independently at the full-text level by two reviewers (N.A. and A.A.). Disagreements between reviewers were resolved by discussing and including a third reviewer (K.A.). Population, Intervention, Comparative, and Outcomes (PICO) research question design guided the formulation of the research question for the current review, as shown in Table 1.

**Table 1:** PICO's statement for the systematic literature search

Systematic literature search	
Population	Pregnant women
Intervention	The use of selective episiotomy
Comparison	The use of routine episiotomy
Outcome	Perineal tears, Apgar score, admission to the NICU, blood loss, pain scores

Eligibility criteria

Inclusion criteria were limited to studies using randomized-controlled trials (RCT) and case-control studies design that compares routine episiotomy to selective episiotomy during vaginal births, studies published in English. Birth outcomes evaluated in this review were perineal tears, blood loss, Apgar score, pain scores, and admission to NICU. The search was also limited to studies published between September 2016 and August 2023.

Data extraction and synthesis

Two reviewers (N.A. and A.A.) independently extracted the eligible studies' data. Data were extracted using a developed form that recorded the study characteristics, including the study aim, sample size, and birth outcomes. Information related to the randomization and allocation process was also extracted to ensure the validity of studies and minimize biases. The primary outcomes assessed in the studies included in this review were minor perineal tears, severe perineal tears (comprising third and fourth-degree tears), and obstetrical anal sphincter injuries, collectively classified as severe perineal trauma. Additionally, the health status of newborns was evaluated through Apgar scores, admission to the Neonatal Intensive Care Unit (NICU), and instances of fetal distress. Maternal outcomes encompassed measuring blood loss during and post-labor and pain scores. We considered other outcomes encompassing skin lacerations that did not need suturing, time of repair for tears (excluding time for episiotomy repair), and maternal pain related to the perineal area.

Data synthesis in this review involved narrative synthesis. Narrative synthesis involves summarizing the results qualitatively, highlighting the differences in primary and other outcomes between the routine and selective episiotomy groups. This approach offered a thorough overview of the evidence by detecting the variation across studies. The clinical approach to heterogeneity involves evaluating the variations in study characteristics, interventions, and populations. Heterogeneity was transparently reported in the results section to ensure the validity and reliability of the findings [18].

Study selection

**Figure 1:**
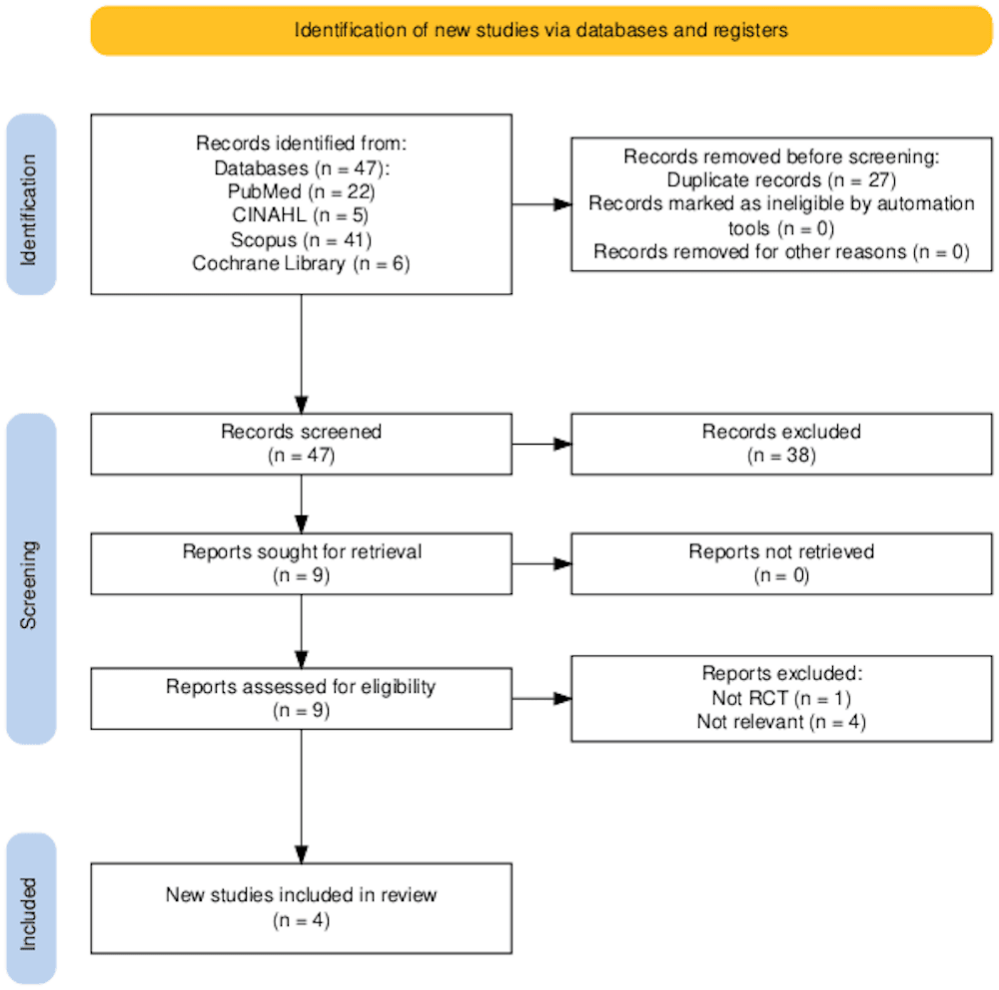
Preferred Reporting Items for Systematic Review and Meta-Analyses (PRISMA) flowchart for the selected studies

The search yielded 121 studies. Forty-seven studies remained for screening after we excluded duplication. We were screening abstracts produced by nine eligible studies. Five studies were excluded because they did not fulfill the inclusion criteria. Finally, four studies remained for narrative synthesis [19-22], as presented in Figure 1.

Reporting of bias and certainty of findings assessment

The JBI Critical Appraisal Checklist for Randomized Controlled Trials was used to evaluate the quality of the included studies [23]. Two reviewers (N.A. and K.A.) independently assessed the quality of the studies, and a third reviewer (A.A.) intervened to resolve any disagreement. According to methodological quality, studies were classified as low, moderate, and high risk of bias involving the appraisal of the randomization process, allocation concealment, blinding of assessors, researchers, and participants, and any other risk of bias. This categorization is a general assessment without specific cut-off points (Table 2).

**Table 2:** JBI critical appraisal checklist for randomized control trials for included studies JBI - Joanna Briggs Institute

	Atef et al., 2022 [20]	Thakur et al., 2020 [21]	Sangkomkamhang et al., 2020 [19]	Ahmed et al., 2018 [22]
True Randomization	Yes	Yes	Yes	Yes
Allocation Concealment	Unclear	Unclear	Yes	Unclear
Baseline Similarity	Yes	Yes	Yes	Yes
Participant Blinding	NA	NA	NA	NA
Treatment Delivery Blinding	NA	NA	NA	NA
Outcome Assessment Blinding	Unclear	Unclear	Unclear	Unclear
Treatment Group Consistency	Yes	Yes	Yes	Yes
Follow-Up Completeness	Yes	Yes	Yes	Yes
Intention-to-Treat Analysis	Unclear	Unclear	Yes	Unclear
Uniform Outcome Measurement	Yes	Yes	Yes	Yes
Reliable Outcome Measurement	Unclear	Unclear	Yes	Unclear
Appropriate Statistical Analysis	Yes	Yes	Yes	Yes
Trial Design and Deviations	Yes, with no deviation	Yes, with no deviation	Yes, with no deviation	Yes, with no deviation
Overall Quality Assessment	Moderate risk for bias Moderate-quality evidence	Moderate risk for bias Moderate-quality evidence	Low risk for bias High-quality evidence	Moderate risk for bias Moderate-quality evidence

Findings

In this section, we present the key findings derived from the systematic review comparing selective and routine episiotomy during vaginal birth. The analysis, conducted following the JBI methodology, aimed to shed light on the impact of episiotomy practices on birth outcomes.

Study characteristics

The main characteristics of the significant results of the included studies are shown in Table 3. Mothers' age ranged between 22.82 and 26.7 years. In the first study, Ahmad et al. included 400 nulliparous women; the maternal age in the control group was significantly higher than the interventional group. The justification for applying episiotomy in the interventional group was the high risk for perineal trauma, fetal distress, and maternal exhaustion [22]. The second study was also from Egypt, and the population was 120 nulliparous women [20]. The third study was from India and included 200 nulliparous or secondo-gravida whose perineum was tight (similar to nulliparous perineum characteristics) [21]. Thakur et al. justified the use of episiotomy for the interventional group in cases of shoulder dystocia, rigid perineum, malposition for the head of the baby, breech presentation, and forceps and vacuum deliveries. However, the fourth study, which is from Thailand, included 3006 women without criteria regarding parity. The rationale for selective episiotomy was shoulder dystocia, fetal distress, instrumental vaginal delivery such as using vacuums or forceps, and when perineal tears were imminent [19].

**Table 3:** Main characteristics of the significant results of included studies R.R: Risk Ratio, CI: Confidence Interval, ª: Mean, *: Standard Deviation, **: Chi-square test, ***: t-test, ****:  Significant, ¹: Median

Author & Year & Country	Aim	Design	Major outcomes	Comparative groups		R.R & (95%CI)	P-value	Sample size	
				Control group	Intervention group			Control group	Intervention group
Ahmad et al., 2018 [22]	To compare maternal and fetal outcomes between selective and routine episiotomy groups in the early postpartum period among primiparous.	RCT	Perineal tears**			Not estimated	P < 0.001*****	200	200
			No tear	0%	61%				
			1st-degree tear	11%	12.5%				
			2nd-degree tear	79%	20%				
			3rd-degree tear	3%	1.5%				
			4th-degree tear	0.5%	0%				
			Labial laceration	6.5%	5%				
			Blood loss*** (ML)	623ª ±190.40*	397.55ª±126.42*		P < 0.001*****		
			Apgar at 1 min***	7¹	7¹		0.151		
			Apgar at 5 min***	9¹	9¹		0.158		
			NICU admission (Yes)**	3%	3%		1.000		
Atef et al., 2022 [20]	To assess the effect of the application of routine versus selective episiotomy on mothers and babies	RCT	Perineal tear**	Yes: 11 women (18.3%), No: 49 women (81.7%)	Yes: 18 women (30%), No: 42 women (70%)	Not estimated	0.136		
			Obstetrical anal sphincter injuries**				0.854	60 Nulliparous women	60 Nulliparous women
			3A	1 (1.7%)	3(5%)				
			3B	2 (3.3%)	1 (1.7%)				
			3C	1(1.7%)	1 (1.7%)				
			No	56 (93.3%)	55 (91.7%)				
			Blood loss (ML)***	289.68ª ±98.05*	223.62ª ±111.94*		0.001****		
			Apgar at 1 min***	7.33ª ±0.90*	7.12ª ±0.90*		0.190		
			Apgar at 5 min***	9.52ª ± 0.65 *	9.43ª ± 0.7*		0.500		
Thakur et al., 2020 [21]	To evaluate episiotomy procedure and try to decipher its role in maternal outcomes	Prospective cohort study	Perineal tears **			Not estimated	P ≤ 0.001****	100 primiparous	100 primiparous
			No perineal tear	0 women (0%)	38 women (38%)				
			1st-degree tear	0 women (0%)	25 women (25%)				
			2nd-degree tear	26 women (26%)	90 women (90%)				
			3^rd^& 4^th^ degree tear	Ten women (10%)	Nine women (9%)				
			Perineal trauma (yes)**	100%	62%				
			Pain (presence of mild pain or more according to visual analog scale)**	100%	60%		P < 0.001		
Sangkomkamhang et al., 2019 [19]	To determine whether restrictive or routine episiotomy results in fewer complication among pregnant Southeast Asian women.	A multicenter randomized controlled trial	Intact perineum	2.3%	7.3%	3.12 (2.15–4.53)	P <0.001****	1504 women	1502 women
			Mild perineal tears (1^st^&2^nd^)	94.4%	90%	0.95 (0.93–0.97)	P < 0.001****		
			Severe perineal tears	3.1%	2.2%	0.72 (0.46–1.12)	P = 0.14		
			Additional vaginal laceration	14.8%	30.4%	2.06 (1.78–2.37)	P < 0.001****		
			Prolonged perineal repair times (>30 min)	13.1%	11.6%	0.89 (0.73–1.08)	P < 0.001****		
			Pain at 2 hours (moderate pain or more according to visual analog scale)	55.0%	48.3%	0.88 (0.82–0.94)	P <0.001****		
			Pain after 24 hours (moderate or more)	58.6%	53.4%	0.91 (0.85–0.97)	P <0.001****		
			Blood loss (≥ 500 ml)	1.1 %	1.0%	0.88 (0.44–1.76)	Not reported		
			Apgar score <7	0.7%	0.5%	0.73 (0.29, 1.81)	Not reported		
			NICU Admission (Yes)	4.8%	5.4%	1.12 (0.82, 1.52)	Not reported		

Risk of bias

Regarding risk assessment, all four studies exhibit true randomization processes, which enhance the validity of the results [19-22]. All studies except Sangkomkamhang et al. found allocation concealment unclear. Uncertainty around the blinding of outcome assessment may influence outcome reporting. The reliability of outcome assessment was unclear, although unified outcome measures were applied [23]. To sum up, the Thai study by Sangkomkamhang et al. reported a high level of evidence and a low risk of bias, while other studies by Atef et al., Thakur et al., and Ahmad et al. showed a moderate risk of bias and moderate quality of evidence.

Synthesis of results

In this section, we present our synthesis of results from studies by Ahmad et al., Atef et al., Thakur et al., and Sangkomkamhang et al.; to assess the effectiveness of routine episiotomy versus selective episiotomy on mothers and newborns birth outcomes. Starting with maternal outcomes, Ahmad et al. reported that perineal tears (first, second, third, and fourth degrees) and skin lacerations were lower in selective episiotomy compared to routine episiotomy. Thakur et al. confirmed when they found notable overall lower rates of perineal tears among the selective episiotomy group [21]. However, Atef et al. found no significant differences regarding perineal tears, including obstetrical and sphincter injuries in all degrees (3A, 3B, and 3C), between the selective episiotomy group and the routine episiotomy group [20]. Also, Sangkomkamhang et al. reported no significant differences between the two groups regarding severe perineal tears, so restrictive episiotomy guaranteed more intact perineum than the routine policy of episiotomy. Regarding skin lacerations, restrictive episiotomies had a significantly higher risk than routine episiotomies, but this did not increase the time for suturing [19].

Blood loss estimation was significantly lower among the selective episiotomy group [20,22], while Sangkomkamhang et al. found no significant differences between groups concerning risk for postpartum hemorrhage [19]. Thakur et al. had not studied this outcome [21]. Sangkomkamhang et al. offered insights into pain rate; they found that the experienced pain in the perineal area was less recurrence among the selective episiotomy group at 2 and 24 hours [19]. Supported by Thakur et al., they found that 100% of the control group experienced mild pain or more, while 60% of the study group had experienced mild pain or more (P < 0.001) [21]. The combined evidence indicates that the adoption of selective episiotomy practices, when parallel with the routine episiotomy approach, may contribute to a reduced frequency of postpartum pain.

Moving to fetal outcomes, the Apgar score at one and five minutes had no significant differences across studies [19-22]. Admission to the NICU was higher among the selective episiotomy group, although Apgar scores were similar between the two groups [22]. In the context of the studies of Ahmad and Sangkomkamhang et al., it seems there is agreement. These studies show no statistically meaningful divergence in NICU admissions between the group that received routine episiotomy and the group where episiotomy was selectively performed [19]. Consequently, these research outcomes imply a shared standpoint that selective episiotomy is not associated with an elevated likelihood of NICU admissions compared to routine episiotomy.

Discussion

In this review, we evaluated the impact of applying the routine episiotomy policy compared to the selective episiotomy policy for mothers and newborns. The included studies examined a range of maternal and neonatal factors among the routine episiotomy group and the selective episiotomy group. The primary outcomes were perineal tears, blood loss, pain scores, and neonatal assessment such as Apgar score and admission to the NICU-other outcomes, such as time of repair.

The data pooled from all studies provided an overview of the impact of the application of episiotomy policy on maternal outcomes. Ahmad et al. found that second and third degrees of tears were lower among the selective episiotomy group than the routine group [22]. Nevertheless, Atef et al. found that perineal tears and obstetrical anal sphincter injuries had no significant differences between the selective versus the routine group of episiotomy. The third and fourth degrees of perineal tears were notably higher among the routine group than the selective episiotomy group [20]. To conclude, the episiotomy group had a higher overall rate of perineal tears and the need for suturing compared to the control group.

According to blood loss, the selective group of episiotomies had lower rates of blood loss compared to the routine group [20, 22]. Sangkomkamhang et al. reported no significant differences between groups. Therefore, the selective episiotomy policy resulted in a lower rate of maternal blood loss, or it did not exceed the rate of blood loss of the routine episiotomy policy [19]. The pain was studied by Sangkomkamhang et al., and they found that the selective episiotomy group had lower pain scores at two and 24 hours compared to the routine group [19]. Also, Thakur et al. found significantly lower pain scores in the study group than in the control group [21]. This suggests that the selective policy of episiotomy may contribute to enhancing pain management methods and provide comfort to mothers after birth.

Ahmad et al., Atef et al., and Sangkomkamhang et al. investigated fetal outcomes. They reported similar Apgar scores at one and five minutes and admission to the NICU between the two groups [19,20,22]. Sangkomkamhang et al. found no differences in Apgar scores at one and five minutes between groups; however, admission to the NICU was higher among the selective episiotomy group, but they justified that selective episiotomy according to intervention criteria was done just in cases of fetal distress and maternal exhaustion. They suggested that the selective episiotomy policy had no significant negative impact on newborns.

Limitations and implications

Although the included studies highlight the effectiveness of routine versus selective episiotomy on mothers and newborns, we should acknowledge several limitations. Firstly, we noted a significant heterogeneity regarding the definition of maternal and neonatal outcomes across studies. We faced challenges regarding variations in study participants’ inclusion criteria, which limited the comparison between studies. Also, four studies were from different cultures, meaning variations in cultural practices in healthcare settings may impact outcomes.

The results of the current review shed light on the advantages of consuming selective episiotomy policy among mothers and newborns; it can reduce the risk of perineal trauma, especially third and fourth degrees, including obstetrical anal sphincter injuries. Variations in study methodologies, such as inclusion criteria and study samples, may limit the evidence pooled from studies. We recommend further studies with standardized methodologies to provide robust findings regarding overall evidence.

## Conclusions

This systematic review compared the maternal and newborn outcomes of routine versus selective episiotomy during vaginal births. Some studies indicate selective episiotomy is associated with a lower incidence of perineal tears, including third and fourth degrees. In contrast, other studies supported the idea that restrictive episiotomy guaranteed more intact perineum than the routine policy of episiotomy because they did not find significant differences regarding the incidence of perineal tears-also, selective use of episiotomy associated with lower rates of blood loss and less pain. Regarding Apgar scores, there were no significant differences between routine and selective episiotomy groups. Admission to NICU was slightly higher among the selective episiotomy group, likely related to specific criteria of intervention to apply episiotomy in cases of fetal distress.

In conclusion, the current review limits the assumptions of the continued need for routine episiotomy to benefit from its effects on mothers and newborns. It presents selective episiotomy as an appropriate alternative in urgent cases, as we demonstrated its positive impact on mothers and newborns.
