# Correction: A Systematic Review of the Use of Routine Versus Selective Episiotomy for Vaginal Birth

**DOI:** 10.7759/cureus.c304

**Published:** 2025-09-23

**Authors:** Nour A Alrida, Amal Ababneh, Khawla Al-Sharif, Diana Arabiat, Jafar Alshraidah, Basheer Al-Zu'bi

**Affiliations:** 1 Nursing, Yarmouk University, Irbid, JOR; 2 Nursing, Jerash University, Jerash, JOR; 3 Critical Care, Jordanian Ministry of Health, Amman, JOR; 4 Maternal and Child Health Nursing, Faculty of Nursing, University of Jordan, Amman, JOR; 5 Clinical Research and Innovation, School of Nursing and Midwifery, Edith Cowan University, Joondalup, AUS; 6 Nursing, University of Jordan, Amman, JOR; 7 Critical Care, Irbid University College, Al-Balqa Applied University, Al-Salt, JOR

This article has been corrected to fix the following typo in the PRISMA diagram: The number of reports excluded was originally stated as 6. This has now been corrected to 5.

